# Amyotrophic onset in GCH1 dopa-responsive dystonia

**Published:** 2019-10-07

**Authors:** Seyed Amir Hasan Habibi, Alberto Albanese, Antonio E. Elia, Paria Arfa-Fatollahkhani, Neda Hashemi

**Affiliations:** 1 Department of Neurology, Rasoul-e-Akram Hospital, Iran University of Medical Sciences, Tehran, Iran; 2 Department of Neurology, Humanitas Research Hospital, Rozzano, Milan, Italy; 3 Fondazione I.R.C.C.S. Istituto Neurologico Carlo Besta, Milan, Italy; 4 Department of Neurology, Iran University of Medical Sciences, Tehran, Iran; 5 Department Obstetrics and Gynecology, Rasoul-e-Akram Hospital, Iran University of Medical Sciences, Tehran, Iran

**Keywords:** Dystonia, Dopa-Response Dystonia, Atrophy

Dopa-responsive dystonia (DRD) belongs to combined dystonia syndrome (dystonia-plus syndrome)^1^ which encompasses non-degenerative and neurometabolic disorders characterized by combination of dystonia as the prominent sign, with another movement manifestation. Parkinsonism and myoclonus are the main disturbances accompany dystonia in the combined dystonia syndrome. Dystonia with parkinsonism includes DRD [DYT5, tyrosine hydroxylase (TH), and sepiapterin reductase (SPR)], dopamine agonist-responsive dystonia, rapid-onset dystonia parkinsonism (DYT12), and early-onset dystonia with parkinsonism (DYT16). However, dystonia combined with myoclonus is just classified as myoclonus dystonia (DYT11).^2^ DRD can be inherited in either autosomal dominant or autosomal recessive patterns. The autosomal dominant inheritance results in the typical phenotype of DRD, known as DYT5 or Segawa disease, which is caused by heterozygous mutations of guanosine triphosphate (GTP) cyclohydrolase I gene (GCH1). Mutations of the TH and SPR genes are responsible for autosomal recessive types of DRD.^3^

DYT5 is represented as progressive lower limbs dystonia with childhood-onset at the common age of 2-5 years. It shows diurnal fluctuations, which are aggravated toward the evening and alleviated by sleeping. Excellent and sustained response to the low dose of levodopa is the marked feature of DYT5 disease. Additional parkinsonism and spasticity may present later in life.^4^

Moreover, hemiatrophy of the brain, body, or both has been reported in patients with DRD, associated with a biochemical lesion located in basal ganglia.^5^ However, focal atrophy and muscle weakness rarely accompanies DRD. Interestingly, we aimed to introduce weakness and focal muscle atrophy as the onset manifestations of DRD in an elderly man misdiagnosed for about 70 years.

Proband was an Italian 78-year-old right-handed man born in 1935.

He passed an uneventful childhood with normal developmental milestones of standing, walking, and speaking. His health condition remained well until the age of 10; when he was limited in competing with his sport playmates due to some walking problems. As the problems progressed, left leg weakness (-4/5) with brisk reflexes and progressive atrophy of anterior calf muscles (Figure 1) were presented in 1973, at the age of 38. Hence, some workups were undertaken; biopsy of left tibialis anterior muscle revealed neurogenic atrophy. Electromyography (EMG) and nerve conduction velocity (NCV) evaluation also reported neurogenic pattern and motor sensory axonal polyneuropathy of this muscle. 7 years later, he started to develop asymmetric rest dystonia which was accompanied with tremor in his lower limbs, dominantly in the left side. Thereafter, low dose of levodopa [250 L-dopa-equivalent daily dosage (LEDD)] was administrated for the him, and an excellent and sustained response was shown. Notably in 1990, besides these neurologic impairments, he suffered from a peptic ulceration and underwent a gastric resection surgery.

**Figure 1:**
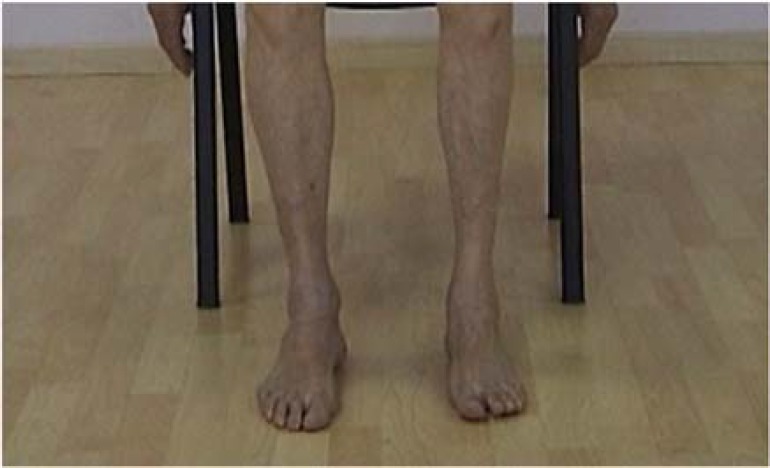
Left tibialis anterior muscle atrophy

In 2013, after taking levodopa for 33 years, there was no motor complications, and no progression was detected in his muscle atrophy. Overall, in the examinations, the cranial nerves functions were normal; and just delayed saccadic eye movements were detected. In addition to the lower limbs dystonia, he had mirrorism and extensor dystonia of the upper extremities digitrums. In accordance with the para-clinic assessments, DaTscan was normal, and brain magnetic resonance imaging (MRI) revealed mild bilateral T2 hypointensity in lentiform nuclei, which was suspected to be in accordance with senile brain atrophy process. 

MRI of lumbar spine was also applied; L3-L4 disk bulging without any spinal cord compression or lesion was reported. Laboratories assessments including routine blood tests, thyroid function test, levels of vitamin B12, B6, B2, and E as well as folate level in blood were done, and all the results were in normal limits.

Interestingly, genetic analysis revealed pathogenic mutation IVS5+1 in the GCH1 gene, which is known as the classic form of autosomal dominant DRD (DYT5). In this regard, Proband’s daughter experienced dystonic postures of her left leg at the age of 8. Soon after, she developed writing dystonia with diurnal fluctuations. Dystonia progressed in generalized form. Therefore, at the age of 30, L-dopa/Carbidopa (2 mg/kg per day) was started and resulted in marked and persistent improvements of dystonia. Genetic test also confirmed inherited pathogenic mutation IVS5+1 in the gene GCH1 (DYT5).

In this case, DRD represented as neuropathy and weakness with the clinical manifestation of muscle atrophy. Within the long follow up time, no additional parkinsonism symptoms or generalized dystonia and levodopa complications were detected in the patient. Scarce data are available suggesting limb atrophy and weakness as the presenting features of DRD disease; and there are case reports of DRD and hemi-atrophy of brain, bone or combination of both,^5^ but in this patient the muscle atrophy was due to neuropathy as a presenting feature. Thereafter, as the atrophy and weakness started at the age of ten, neither gastric surgery nor levodopa side effects could be responsible for the patient neuropathy and clinical features. Lumbosacral MRI result, brisk reflexes, and EMG-NCV report also roll out the structural abnormalities and anterior horn cell diseases as the etiology of limb atrophy, respectively. Importantly, Proband’s daughter developed writing dystonia and generalized form of dystonia at last, which suggests that intrafamilial variability of clinical features should be considered in the patients with DRD.

The present case highlights that muscle atrophy and weakness can be the first manifestations of DRD, which may result in misdiagnosis and mismanagement of the patients for several years.

## Notes:

**How to cite this article:** Habibi SAH, Albanese A, Elia AE, Arfa-Fatollahkhani P, Hashemi N. Amyotrophic onset in GCH1 dopa-responsive dystonia. Iran J Neurol 2019; 18(4): 181-3.

## References

[1] Albanese A, Bhatia K, Bressman SB, Delong MR, Fahn S, Fung VS (2013). Phenomenology and classification of dystonia: a consensus update. Mov Disord.

[2] Fahn S, Jankovic J, Hallett M (2011). Principles and practice of movement disorders.

[3] Albanese A, Jankovic J (2012). Hyperkinetic movement disorders: Differential diagnosis and treatment.

[4] Edwards M, Quinn M, Bhatia K (2008). Parkinson's disease and other movement disorders.

[5] Greene PE, Bressman SB, Ford B, Hyland K (2000). Parkinsonism, dystonia, and hemiatrophy. Mov Disord.

